# Epidemiology and Risk Factors of Dry Eye Disease: Considerations for Clinical Management

**DOI:** 10.3390/medicina60091458

**Published:** 2024-09-05

**Authors:** Alexis Ceecee Britten-Jones, Michael T. M. Wang, Isaac Samuels, Catherine Jennings, Fiona Stapleton, Jennifer P. Craig

**Affiliations:** 1 Department of Optometry and Vision Sciences, Faculty of Medicine, Dentistry and Health Sciences, The University of Melbourne, Parkville, VIC 3010, Australia; ac.brittenjones@unimelb.edu.au; 2 Department of Ophthalmology, Aotearoa New Zealand National Eye Centre, The University of Auckland, Auckland 1023, New Zealand; mwan759@aucklanduni.ac.nz (M.T.M.W.); isaac.samuels@middlemore.co.nz (I.S.); c.jennings@auckland.ac.nz (C.J.); 3 School of Optometry and Vision Science, Faculty of Medicine and Health, UNSW Sydney, Sydney, NSW 2052, Australia; f.stapleton@unsw.edu.au

**Keywords:** dry eye disease, blepharitis, ocular surface, epidemiology, risk factors, management considerations

## Abstract

Dry eye disease is a multifactorial condition characterised by tear film instability, hyperosmolarity and ocular surface inflammation. Understanding the epidemiology of dry eye disease and recognising both modifiable and non-modifiable risk factors can assist eye care practitioners in assessing, treating, and managing patients with the condition. This review considers current knowledge surrounding its incidence and prevalence, as well as associated demographic, systemic, ocular, and iatrogenic, and lifestyle-related modifiable risk factors. Population-based prevalence estimates vary according to the diagnostic criteria used to define dry eye disease, as well as severity and demographic characteristics of the population. Considering recent data and variable population demographics, conservative prevalence estimates suggest that 10–20% of the population over 40 years of age report moderate to severe symptoms and/or seek treatment for dry eye disease. Individuals with specific non-modifiable demographic risk factors may be at increased risk of developing dry eye disease. Advanced age, female sex and East Asian ethnicity have been identified as key non-modifiable demographic features predisposing individuals to dry eye disease. Systemic conditions that have been associated with an increased risk of dry eye disease include migraine, Sjögren syndrome, connective tissue disorders, mental health disorders, diabetes mellitus and androgen deficiency. Medications that may contribute to this risk include antidepressants, antihistamines, and hormone replacement therapy. Ocular and iatrogenic risk factors of dry eye disease include blepharitis, *Demodex* infestation, ocular surgery, blink completeness, contact lens wear, and topical ophthalmic medications. A range of modifiable lifestyle factors that can increase the risk of dry eye disease have also been identified, including low humidity environments, digital screen use, quality of sleep, diet, and eye cosmetic wear. Dry eye is a common disease affecting millions globally. Increasing knowledge regarding its associated risk factors can better prepare the eye care practitioner to successfully manage patients with this ocular surface disease.

## 1. Introduction

Dry eye disease is a complex multifactorial condition that is characterised by homeostatic disturbances of the ocular surface and tear film [1]. Aetiologically, dry eye disease is highly heterogenous and diverse, with multiple causes and risk factors identified as having the potential to impact different components of the lacrimal functional unit [1,2]. Nevertheless, the condition is typically classified into two broad subtypes, aqueous deficient and evaporative dry eye disease, which, respectively, represent insufficient production or accelerated evaporative losses of the tear film [1,3]. Regardless of the aetiological cause, however, a vicious circle of tear film instability, hyperosmolarity, and inflammation is triggered, which can culminate in progressive damage to the ocular surface and lead to neurosensory abnormalities, both of which in themselves further propagate the cycle [3,4,5].

Dry eye disease is associated with a myriad of ocular symptoms, including grittiness, epiphora, photosensitivity and transient visual disturbance [1,2,5]. At an individual level, the clinical symptoms of dry eye disease are recognised to have significant adverse effects on ocular comfort, visual function, work productivity, and quality of life, and can impair the functional ability to perform tasks of daily living, such as reading, digital screen use, driving, and outdoor recreational pursuits [2,6,7,8]. At the population level, dry eye disease is associated with a profound public health and financial burden, with reports from 2011 estimating that around US $3.8 billion per year is spent on the treatment of the condition in the United States [9]. Moreover, when other associated costs are accounted for, including physician visits and productivity loss, the total societal cost even over a decade ago was estimated to be US $55.4 billion per year in the United States [9]. Furthermore, the global burden of dry eye disease is projected to increase, on account of the ageing population and the increasingly pervasive use of digital screens among paediatric and young adult populations [2,10,11,12].

**Figure 1:**
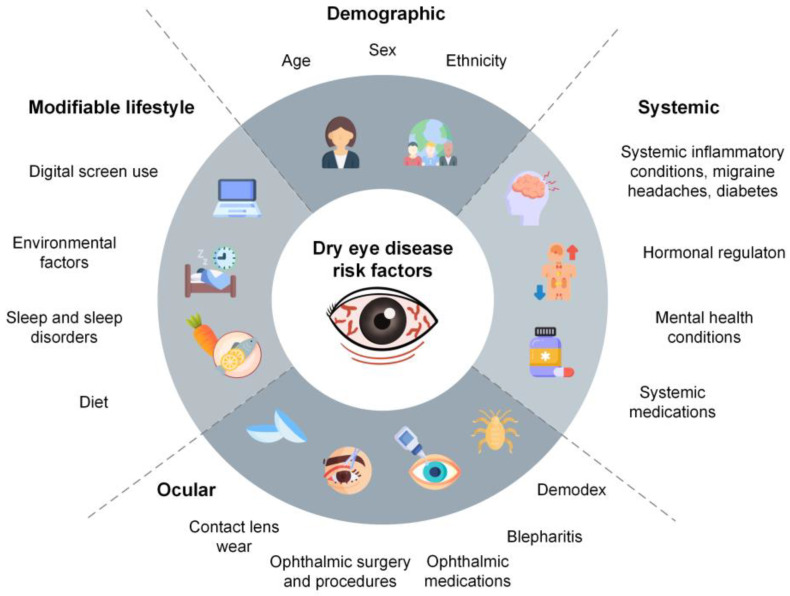
Risk factors for dry eye disease discussed in the present review, including demographic, systemic, ocular, and modifiable risk factors, as reported in the literature.

The 2023 Tear Film and Ocular Surface Society (TFOS) Lifestyle Workshop Reports highlighted a number of everyday lifestyle factors that can impact the onset and severity of ocular surface disease [13]. While treatments can help relieve dry eye symptoms, epidemiological research is important in the clinical management of dry eye disease for a number of reasons. Firstly, the identification of modifiable and non-modifiable risk factors can assist eye care practitioners in assessing, treating, and managing patients with the condition (Figure 1) [2,14]. Secondly, an understanding of the epidemiological patterns of dry eye disease prevalence can inform the development of more cost-effective population-based preventative strategies, including targeted screening, risk factor modification, and health promotion interventions [2,15]. Thirdly, characterisation of the prevalence and incidence of the condition enables recognition of the magnitude of the associated public health burden and can assist in advocating for increased funding for treatments at both individual and population levels, as well as investment into research and development of novel therapies [2].

The purpose of the current review is to provide an overview of the existing literature (searched up to 31 May 2024) on the epidemiology of dry eye disease, including modifiable and non-modifiable risk factors, to support clinical management.

## 2. Prevalence

Population-based prevalence estimates for dry eye disease vary significantly based on disease definition, severity, and demographic characteristics of the population [2]. This overview focuses on population-based studies involving more than 500 participants and excludes publications with a limited age range of participants or those restricted by occupation. The search criteria have been reported previously [2]. Many studies evaluating the prevalence of dry eye disease have been performed in Caucasian and East Asian populations, with a paucity of data in most other regions [16].

Several large population-based studies using the Women’s Health Study criteria, arguably capturing more severe disease (severe symptoms of dryness or irritation constantly or often, and/or a prior diagnosis of dry eye disease by a clinician) have reported age-adjusted prevalence rates of 4.3% (males only) to 12.8% (both sexes) in Caucasian populations [17,18,19,20,21] and 16.0% to 23.7% in East Asian populations [22,23,24]. Prevalence assessed using the Salisbury Eye Evaluation instrument in Singapore was 8% [25] and incidence, 5.1%, per year [26]. Female sex and older age have been consistently identified as risk factors in prevalence studies using symptoms-based criteria or a prior diagnosis of dry eye made by a clinician [19,27].

The majority of published studies, however, have explored rates of disease in the over 40s population. Using the Women’s Health Study criteria [28], the Lifelines cohort study in the Netherlands established an overall prevalence of dry eye disease of 9.1% [20], noting that prevalence increased with age, particularly in individuals above 50 years, and women exhibited twice the rate of disease than men in this study. However, in this study, individuals aged 20–30 years had a similar rate of disease to those aged 40–50 years. Similarly, the National Health and Wellness Survey in the USA examined rates of diagnosed dry eye disease and undiagnosed but symptomatic disease and found a combined prevalence of 9.3%. This study also found the prevalence of diagnosed dry eye disease to be similar among the 18–50-year-old age groups [19]. The high rates of disease in younger adults are speculated to arise as a consequence of greater exposure to contact lens wear and/or digital device use.

The prevalence of dry eye disease characterised by both signs and symptoms has been reported as 8.7–11.0% in Caucasians [29,30,31,32], 16.7–33.4% in East Asian populations [22,33,34,35,36,37], and 26.2% in India [38]. In an older Caucasian population (over 65 years), the prevalence was 21.4% [39]. Limited studies have been carried out in Africa, and one small community study (n = 363) has estimated signs and symptoms of dry eye to be present in 32.5% of residents in Southwest Nigeria [40].

Signs of meibomian gland dysfunction (MGD) are present in 30–35% of Caucasian individuals [29,30,41,42] and in 33–50% of East Asian individuals [33,37,43,44], rising to 51.8–60.8% in the over 65 age group [43,44,45]. In an Iranian population-based study, the prevalence of MGD was 26.3% in an adult population [46] rising to 71.2% in the over 60s age group [47]. It is recognised, however, that up to two-thirds of those with the disease may be asymptomatic [41] and that age-related lid and gland changes underpin high rates of asymptomatic disease in older adults. A systematic review of inter-ethnic disparities in the natural history of dry eye disease suggested that meibomian gland changes were apparent earlier in life in East Asian eyes than Caucasian eyes [48]. Compared with studies exploring signs and symptoms of dry eye, several studies have suggested a higher prevalence of MGD, specifically, in males (up to 2.5 times) compared with females [37,47].

A recent systematic review found an inverse relationship between dry eye prevalence and Gross National Income (GNI), with a progressive decline from lower-middle-income to upper-middle-income to high-income countries [49]. However, this finding may also be confounded by access to services, education, lifestyle, and societal factors [50].

In summary, as a conservative estimate, 10–20% of the population over 40 report severe symptoms and/or seek treatment for dry eye disease. Irrespective of the definition of disease, the prevalence consistently varies with age and race, although the impact of sex on MGD is equivocal. Importantly from a public health and resource planning perspective, there is some evidence from a recent audit of medical claims for the disease prevalence having increased in a US population over time [51].

There remains a paucity of appropriately powered population-based studies in certain regions. Given the reports of dry eye in younger age groups in East Asian and Caucasian populations, appropriately powered studies in youth and adult populations below 40 years are required to address the prevalence of dry eye disease and major risk factors in this group.

## 3. Demographic Risk Factors

### 3.1. Age

Epidemiological studies have consistently demonstrated a positive association between advancing age and the development of dry eye disease, irrespective of ethnicity [2,11,12,19,52]. A number of population-based cross-sectional studies have found that the prevalence and severity of clinical signs and symptoms of dry eye disease increases with ageing [2,11,12,19,52]. Indeed, dry eye disease is recognised to be a complex and multifactorial degenerative condition from progressive exposure to a myriad of physiological and environmental factors that can trigger changes to systemic hormonal and neurosensory regulation, tear film homeostatic disturbances, and ocular surface inflammatory pathways [2,3]. Paediatric populations generally had lower rates of diagnosed dry eye compared to adults, but symptoms were frequently reported, especially among young females [53]. Key aetiologies responsible for DED in the paediatric population include MGD, autoimmune diseases, and vitamin A deficiency [54], but screen time [55], contact lens wear [56], and other environmental factors could also play a role [53].

Recent studies investigating the natural history of dry eye disease in adults have noted that signs of MGD and evaporative dry eye disease tend to develop at an earlier age than signs of aqueous tear deficiency [12,48]. Moreover, a delay of up to one decade is apparent between the development of signs of MGD, such as gland dropout, and other clinical markers of dry eye disease [12].

The apparent delay between the appearance of markers of MGD and the development of other clinical signs potentially represents a degree of functional reserve at the ocular surface, and a window of opportunity for preventative interventions during the early development of the condition. Nevertheless, it is acknowledged that, to date, there is a paucity of large, population-based, prospective longitudinal studies investigating the association between ageing and the incidence of dry eye disease. Further research in this area is required to corroborate the findings reported in existing cross-sectional epidemiological studies [2].

### 3.2. Female Sex

The stronger predilection towards the development of dry eye disease in females has also been reported by a number of population-based, cross-sectional studies [2,19,57,58]. The association between female sex and dry eye disease is thought to be partially mediated by the regulatory effects of sex steroids, and hypothalamic–pituitary and thyroid hormones on the lacrimal functional unit and immune system [2,57,58]. In addition, the modulatory actions of the sex chromosome complement, sex-specific autosomal factors, and epigenetics [53] are also hypothesised to play a role [57].

### 3.3. Genetics

The involvement of genetic risk factors in dry eye disease is complex. Twin studies suggest that genetic factors moderately contribute to dry eye, with symptoms having a heritability of approximately 30% and signs showing a heritability range of 25% to 80% in a group of British middle-aged and elderly female twins [59]. Several genome-wide association studies (GWAS) have been undertaken to investigate genetic risk factors of dry eye disease [60,61]. The need for precise population stratification, accurate phenotyping, and consideration for environmental and lifestyle risk factors can pose challenges in identifying genetic risk markers that contribute to the susceptibility of developing dry eye disease [62]. More recently, a GWAS, using data from the Taiwan Biobank, identified 11 potential independent risk loci for dry eye disease [60]. None of the top single nucleotide polymorphisms (SNPs) were located on the sex chromosomes. However, the female-specific GWAS also identified a subset of the genes from the overall GWAS, implying that females could experience a distinct genetic contribution to DED pathogenesis. Nonetheless, the functional significance of many DNA variants associated with dry eye disease is still unknown, and further studies are required to validate these genetic associations [62].

### 3.4. Ethnicity

East Asian ethnicity has been consistently identified as a risk factor for the development of dry eye disease [2,48]. Earlier studies identified a higher prevalence of dry eye disease, and more severe symptoms and signs, among population studies based in East Asia compared to European cohorts [2,34,43]. However, it was acknowledged that the results might be confounded by differences in environmental and climatic exposure, as well as research methodology [2,48]. Recent co-located inter-ethnic comparative studies that control for environmental exposure have corroborated the propensity of dry eye disease development among the East Asian ethnic group [63,64]. The association has been hypothesised to be partially attributable to anatomical differences that result in greater eyelid tension, including the more inferior attachment site of the levator palpebrae superioris aponeurosis, increased axial length, as well as difference in the distribution of orbital connective tissue [48]. Higher levels of eyelid tension can also result in a greater tendency for incomplete blinking [65], which leads to increased rates of meibomian gland dropout and dysfunction [66,67].

## 4. Systemic Risk Factors

Dry eye disease has been associated with a number of systemic conditions and medications, as summarised in Table 1 [2,20,68,69,70,71,72,73]. However, the heterogeneity in study methodology and disease definitions across studies poses considerable challenge for comparing findings from previous literature [2].

### 4.1. Systemic Inflammation

Several of the systemic conditions associated with dry eye disease, including migraine, thyroid disease, and connective tissue disorders, share inflammatory modulation as a feature in their pathophysiology [2,20,68,69,70]. An association between dry eye disease and migraine headaches has been reported in a number of case-control and cross-sectional studies [69,70]. In a systematic review in the 2023 TFOS Lifestyle: Lifestyle Challenges report, a meta-analysis pooling data from 11 observational studies showed that the odds of dry eye disease were 1.61 times (95% CI 1.39–1.87) higher in people with migraine compared to those without [74]. Both conditions are thought to involve the activation of inflammatory pathways [3,70], with the pathogenesis of migraine headaches involving the plasma extravasation of neurovascular inflammatory mediators and cytokines triggering trigeminal ganglion hypersensitivity [70]. In addition, ocular irritation and reflex tearing secondary to dry eye disease can further exacerbate migraine headaches through hyperstimulation of the trigeminal ganglion [69,70]. 

Dry eye disease has also been associated with connective tissue diseases, Sjögren syndrome, and symptomatic xerostomia [20,76,77]. These systemic autoimmune conditions can cause inflammatory infiltration and structural damage of the lacrimal gland, which leads to aqueous tear deficiency [2,3,76,77]. There has also been increasing reports of an association between dry eye disease and thyroid disorders [20,57,69]. Thyroid hormones exert anabolic effects and promote lacrimal gland activity, and dysfunction of the thyroid gland can, therefore, lead to reduced aqueous tear production [57]. Thyroid eye disease can also cause incomplete lid closure secondary to orbital tissue swelling, inflammation, and proptosis, thereby contributing to the development of evaporative dry eye disease [57,69].

### 4.2. Diabetes

A number of observational studies have reported a positive association between dry eye disease and type I and II diabetes [2,71,78,79,80]. The pathophysiological connection between type I diabetes and dry eye disease is also thought to be partially mediated by antigen cross-reactivity provoking autoimmune destruction of the lacrimal glands [2,57]. In diabetes, poorer glycaemic control and the presence of microvascular complications have been correlated with increased severity of dry eye clinical signs and symptoms [2,78,81,82]. Peripheral neuropathy, the most prevalent complication of diabetes mellitus [83], can also cause corneal nerve loss and impair neurotrophic support, contributing to the development and exacerbation of dry eye disease [84,85,86]. Loss of corneal nerves associated with diabetes can lead to reduced corneal sensitivity and impaired neural regulation of aqueous tear production, increasing the risk of corneal neurotrophic keratopathy in individuals with diabetes, although it has been acknowledged that this might also contribute to masking and subsequent under-reporting of dry eye symptoms [2,57]. 

### 4.3. Androgen Deficiency

Androgen deficiency has been recognised to be a risk factor for the development of dry eye disease [2,57]. Case-control studies have reported a positive association between dry eye disease with congenital androgen insufficiency syndrome and anti-androgen medication treatment [87,88]. Androgens are involved in the modulation of gene expression, protein synthesis, immune activity, and aqueous tear secretion from the lacrimal gland [57]. In addition, androgens also regulate lipid synthesis and suppress keratinisation within the meibomian glands [57]. Androgen deficient states can, therefore, predispose towards the development of both aqueous deficient and evaporative dry eye disease [2,57].

### 4.4. Mental Health Conditions

There is epidemiological evidence that dry eye disease is associated with mental health co-morbidities, including anxiety, depression, and psychological stress burden [6,72,74,89,90,91]. However, caution needs to be applied when interpreting the correlations reported in observational studies, with consideration of the potential confounding effects of systemic medications used to treat mental health conditions, including antidepressants and anxiolytics, which are recognised to be independently associated with dry eye disease [2,73]. Moreover, it has been acknowledged that the associations detected might be multi-directional, and further prospective longitudinal studies are required to determine whether the development and progression of mental health co-morbidities precedes dry eye disease or occurs as a consequence of the symptoms [2,6]. Dry eye disease and mental health conditions are thought to share common aetiological mechanisms involving pathologic neuroplasticity and somatosensory dysfunction, and it remains to be established whether increased pain sensitivity and somatisation could also affect the perception and experience of clinical dry eye symptoms [2,92]. On the contrary, it is not inconceivable for the debilitating symptoms of dry eye disease and its adverse impacts upon quality of life to further exacerbate pre-existing mental health co-morbidities [2,6].

### 4.5. Systemic Medications

A number of systemic medications have been associated with the development and progression of dry eye disease, including antihistamines, antidepressants, anxiolytics, isotretinoin, and hormone replacement therapy [2,73,93,94,95]. Antihistamines, antidepressants, and anxiolytic medications exert antagonistic action to peripheral muscarinic receptors, thereby mediating the reduction of aqueous tear production in the lacrimal glands, as well as the downregulation of mucin output from goblet cells [2,73]. In addition, some antidepressant medications, such as selective serotonin reuptake inhibitors, also increase the availability of serotonin, which is recognised to modulate corneal nerve sensitivity thresholds and the neuronal regulation of lacrimal secretion [93,96,97]. Although the underlying mechanisms are not fully understood, a number of studies have demonstrated that isotretinoin use is associated with increased meibomian gland atrophy and dropout and reduced tear film stability [73,94]. A correlation between hormone replacement therapy use and dry eye disease has been shown in some population-based cross-sectional studies [95,98], as well as in smaller placebo-controlled and uncontrolled clinical studies [99,100]. This relationship is thought to be partially regulated by the inhibitory action of oestrogen on lipid synthesis within the meibomian glands [2,57].

## 5. Ocular Risk Factors

A multitude of ocular diseases, including anterior blepharitis, meibomian gland dysfunction and *Demodex* infestation, have been recognised to be associated with dry eye disease. Ocular procedures and treatments that can elevate the risk of dry eye disease include contact lens wear, ocular surgery, and certain preservative-containing topical ophthalmic medications.

### 5.1. Blepharitis

Blepharitis is a common ocular condition associated with chronic inflammation of the eyelid tissues that affects either or both of the anterior and posterior eyelid lamellae, and involves pathological changes in the eyelid margin, eyelashes and periocular skin, and dysfunction of the meibomian glands associated with gland blockage and tear lipid insufficiency [3,101]. Despite a lack of understanding surrounding the multifactorial pathophysiology of blepharitis, it is believed that raised levels of bacterial colonisation of the eyelid tissues in blepharitis triggers hypersensitivity reactions, stimulating inflammatory cascades on the ocular surface [3,101,102]. Moreover, release of lipolytic exoenzymes from colonising bacterial species further exacerbates pre-existing ocular surface inflammation and destabilises the tear film through the breakdown of superficial lipid layer components [3,103]. 

Ocular *Demodex* infestation has been linked to blepharitis and the development of dry eye disease [104]. Colonisation of eyelash follicles and meibomian glands with *Demodex folliculorum* and *brevis* species is believed to trigger excessive activation of host inflammatory and immune responses via a range of mechanisms, including to chitin (the key constituent of the *Demodex* exoskeleton) and *Demodex* breakdown products [104,105,106,107]. 

In blepharitis, aqueous tear evaporation may be exacerbated by the associated reduction in quantity and quality of meibomian gland secretions, leading to hyperosmolarity, ocular surface inflammation, and tear film instability [3,104,105].

### 5.2. Contact Lenses

The application of a contact lens to the eye physically divides the tear film into pre- and post-lens layers, affecting both tear film composition and biophysical properties [108]. In particular, contact lens wear is recognised to promote tear film thinning and instability [109], as well as reduce tear wettability and spreading [110], and increase tear osmolarity [109,110]. Increased mechanical stress due to compromised lubrication between the contact lens and the eyelid can also induce epitheliopathy at the lid wiper region of the lid margin [111]. The Beaver Dam offspring study found both past and current contact lens wear to be associated with symptoms of dry eye disease [112]. In people under the age of 50, the odds of dry eye disease was 2.39 times higher in current contact lens wearers compared to those who had never worn contact lenses [112]. The Canadian Dry Eye Epidemiology Study found that 50.1% of contact lens wearers reported dry eye symptoms compared to 21.7% of non-contact lens wearers [113]. Notably, there is overlap between symptoms of dry eye and contact lens discomfort in contact lens wearers [114], with dryness and discomfort being key symptoms contributing to the discontinuation of contact lens wear [115].

### 5.3. Ophthalmic Procedures

In corneal and refractive surgery, corneal incisions cause trans-sectional injury to subepithelial corneal nerves. Damage to corneal nerves leads to reduced tear secretion, altered corneal sensitivity, and impaired wound healing [116]. Loss of corneal sensory innervation also reduces the neurotrophic support for epithelial cell proliferation, further disrupting ocular surface homeostasis, and exacerbating pre-existing dry eye disease or meibomian gland dysfunction [75]. The extent of corneal denervation and rate of recovery depends on the technique and procedure used [117]. The number of sub-basal nerve bundles has been found to decrease by more than 90% immediately after laser in situ keratomileusis (LASIK) treatment, and recover to only to half of the pre-treatment value by one year post-surgery [116]. Compared to LASIK, small incision lenticule extraction (SMILE) refractive surgery, which does not require the creation of a flap like LASIK, has been associated with more vision disturbances in the first month post-surgery but less dry eye symptoms after 3 months or more [75,118].

Dry eye disease can also arise from oculoplastic or lid surgery, such as blepharoplasty and ptosis surgery, where changes in lid margin anatomy and the blinking mechanism impact tear film lubrication of the ocular surface. During the blink, lipids and tears from the meniscus are redistributed onto the ocular surface, replenishing tears lost by evaporation [119]. A continuous lipid layer plays an essential role in preventing aqueous tear evaporation [120]; diminished blink quality and blink incompleteness can lead to a vicious cycle of increased tear evaporation, tear film instability, hyperosmolarity, and ocular surface inflammation [3]. Lid surgery can result in malposition of the eyelid or affect eye closure. Resection of the orbicularis oculi muscle can also affect the lid innervation, and lead to incomplete blinking [121], a decreased blink rate and possible lagophthalmos [122], as well as impacts on Riolan’s muscle (pars ciliaris located in the Orbicularis Oculi), leading to ocular surface exposure, increased tear evaporation and tear film desiccation. Surgery can also cause direct damage to meibomian glands, thereby reducing the delivery of meibum to the ocular surface and impairing the tear film lipid layer quality [123,124].

### 5.4. Ophthalmic Medications

There is evidence that topical ophthalmic medications containing preservatives, such as anti-glaucoma therapies containing benzalkonium chloride, exacerbate symptoms and signs of dry eye disease [125,126,127]. Benzalkonium chloride is a commonly used preservative in ophthalmic formulations, but can cause ocular surface goblet cell loss and epithelial damage due to associated toxic and pro-inflammatory effects [124,128]. Additionally, the detergent-like tensioactive effects of benzalkonium chloride are known to compromise the integrity of the tear lipid layer, increasing susceptibility of the ocular surface to desiccation, inflammation and excessive tear evaporation [124,125,126].

Topical nonsteroidal anti-inflammatory drugs (NSAIDs) have been used in moderate-to-severe dry eye to reduce ocular inflammation [129]. However, use of topical NSAIDs has also been reported to reduce corneal sensitivity [130], and there have been sporadic reports of corneal melting and perforation [131]. Thus, most published studies with NSAIDs in dry eye disease have short term durations of less than one month, and NSAIDs should be used with caution, particularly in patients with Sjögren syndrome [14].

## 6. Modifiable Lifestyle Risk Factors

A number of modifiable lifestyle factors have been evaluated for their potential association with dry eye disease, including environmental exposure [132], digital screen use [133], sleep quality [74], diet [134], and periocular cosmetic application [135]. 

### 6.1. Environmental Factors

Environmental factors that can increase the prevalence of dry eye disease include the presence of air pollutants such as nitrogen oxide and smoke [136,137], low humidity [138], and high altitude [136]. In environments with low relative humidity, such as air-conditioned rooms, or outdoor environments with high wind speeds, the increased water vapour pressure gradient between the ocular surface and the surrounding environment results in greater tear evaporation [136,139]. A decrease in relative humidity by 10% was found to increase the rate of tear evaporation by 28–59% [139]. A post hoc evaluation of 535 participants in the Dry Eye Assessment and Management (DREAM) study found that those living in Mediterranean climate zones of the United States, where there is higher relative humidity, had better tear film stability and corneal fluorescein staining scores than those in other United States climate zones [137].

### 6.2. Digital Screen Time

Several studies have demonstrated a strong association between digital screen-time and dry eye disease [56,133,140]. Every one hour per day increase in digital screen time was found to be associated with a 1.14 higher odds of dry eye disease [141]. The rationale behind this association is possibly related to suppression of the spontaneous blink reflex while performing tasks that require significant visual processing, especially tasks with higher cognitive demands [142,143]. As a consequence, reduced blink rate and incomplete blinking causes poor tear lipid layer integrity and tear film instability [143]. Ocular surface symptoms can be further exacerbated by uncorrected refractive error and/or binocular vision anomalies [133].

Chronic reductions in meibum delivery as a result of reduced blinking can also lead to meibum stasis and predispose to meibomian gland dysfunction [144]. Furthermore, when the eye is in a position of relative up-gaze, as may be encountered with the elevated position of desktop computer monitors, tear film instability can be exacerbated due to the greater interpalpebral fissure height and correspondingly larger exposed ocular surface area [145].

### 6.3. Sleep and Sleep Disorders

There is increasing interest in the potential association between sleep and sleep quality, and dry eye disease. A survey of 15,878 adults aged 20 years and older found that, compared to those achieving 6–8 h of sleep per night, those who sleep for less than 6 h per night had between 1.19 and 1.26 higher odds of having symptoms of dry eye disease [146]. Interestingly, sleep quality has also been found to be reduced in people with dry eye disease [147]. Sleep deprivation and poor sleep quality are known to cause various physiological changes within the body. Reduced sleep leads to an increase in stress hormones, including cortisol, adrenalin, and noradrenalin, and reduction in parasympathetic tone [148], leading to reduced lacrimal tear production and tear hyperosmolarity, thereby contributing to the vicious cycle of dry eye disease. Sleep deprivation also leads to a reduction in systemic androgen levels [149], which can downregulate meibomian gland secretions [87,88]. In addition, sleep deprivation is thought to activate the hypothalamic–pituitary–adrenal axis and alter the circadian rhythm of the renin–angiotensin–aldosterone system hormones, inducing excess diuresis and natriuresis [150]. Together, these changes in systemic hormone levels and relative dehydration can further reduce aqueous tear production.

### 6.4. Diet

Diet is a modifiable risk factor that can influence ocular surface health and dry eye disease. Vitamin A deficiency is associated with xerophthalmia, a severe form of ocular surface disease which can cause conjunctival and corneal xerosis, Bitot’s spots, and keratomalacia [151]. Dietary vitamin A deficiency is a major problem in developing countries and is the world’s leading cause of preventable blindness in children [152]. Other possible causes of vitamin A deficiency include fat malabsorption, liver disorders, and cystic fibrosis [153,154]. 

A low dietary intake of omega-3 fatty acids relative to omega-6 fatty acids has also been found to be associated with an increased risk of dry eye disease [155,156]. The U.S. Women’s Health Study of 39,876 respondents found an omega-6 to omega-3 dietary ratio of 15:1 to be associated with a 2.5-times higher incident risk of dry eye disease relative to a ratio of 4:1 [156]. Increasing dietary intake of omega-3 fatty acids is a strategy for reducing ocular surface inflammation by promoting the production of anti-inflammatory mediators [157,158,159]. Results from a Cochrane systematic review of randomised controlled trials indicate a possible role for omega-3 supplements in the clinical management of dry eye disease [160]; however, there is a lack of certainty in the current evidence concerning the optimal dosage and composition of supplements containing fatty acids to confer the most benefit [161].

### 6.5. Cosmetics and Cosmetic Procedures

An association between periocular cosmetic product application and the development of dry eye disease has been recognised, with multiple accounts of tear film contamination during routine ocular surface examination [135,162,163]. Factors such as eye rubbing, misapplication, passive migration of cosmetic eye products, and accidental application directly to the interpalpebral ocular surface result in tear film contamination [164]. In addition to benzalkonium chloride (as discussed in Section 5.4) [165], other ingredients found frequently in make-up that are toxic to the ocular surface integrity include chlorphenesin, formaldehyde-releasing compounds, parabens and phenoxyethanol [135,166,167,168]. These ingredients are often found in creams, and eyelash glue. Eyelash extensions can also cause ocular surface inflammation and keratoconjunctivitis due to invasion of glue or removal [169]. Retinoids (vitamin A derivatives), often used in anti-ageing serums, are also toxic to meibomian glands [170].

Tear film contamination from eye cosmetics crossing the eyelid margin may result in ophthalmic complications, such as posterior blepharitis, tear film instability, conjunctival pigmentation, ocular surface irritation, and keratitis [135]. It is speculated that mechanisms leading to these complications include debris within the tear film lipid layer, meibum contamination and mechanical meibomian gland blockage [162], which contribute to reduced tear film stability and excessive tear evaporation.

Injection of botulinum toxin is a common elective cosmetic procedure; applications of botulinum toxin to the medial upper and lower eyelid have also been used for treating dry eye symptoms and signs [171,172]. The toxin is thought to inhibit the orbicularis muscle pump medially, promoting tear retention. However, tear film instability after botulinum toxin injections has also been reported [173,174], with higher doses of botulinum toxin seemingly associated with greater effects. The long-term adverse effects of repeated botulinum toxin injections on ocular surface integrity are not known.

## 7. Conclusions

Dry eye disease is a complex and multifactorial degenerative condition with impacts at both an individual and societal level. From individual complaints of ocular discomfort and intermittent visual disturbance to the economic and public health burden, dry eye disease is an ongoing and growing international health issue. Conservatively, 10–20% of the population over 40 are currently estimated to have dry eye disease, and it is recognised to be a worsening public health issue, with an increased prevalence related to the current worldwide ageing population, and in younger adults from contact lens wear and digital device use. Future studies encompassing diverse geographical regions, populations, and varying age groups, particularly younger cohorts, could offer a more comprehensive understanding of dry eye disease epidemiology. Adopting consistent and standardised diagnostic criteria, such as the TFOS DEWS II diagnostic criteria [1], would further improve the reliability of prevalence assessments.

From the current evidence base, there is a clear need for further prospective longitudinal studies to determine whether the development and progression of ocular and systemic co-morbidities precede dry eye disease or occur as a consequence of the condition. Greater consistency in study methodology and disease definitions across epidemiological studies is needed to enable better cross-study comparisons, and to assess the strength of association between dry eye disease and aetiological risk factors. Other areas for evaluation include the role of genetics and epigenetics in the susceptibility to dry eye disease and its subtypes. Nonetheless, the consistent positive association shown between dry eye disease and ocular and systemic risk factors indicate that patients with noted conditions should be carefully screened for dry eye disease.

In conclusion, dry eye disease is a common, yet complex ocular surface condition associated with a myriad of modifiable and non-modifiable risk factors. An understanding of these risk factors provides contextual background to eye care practitioners involved in the assessment, treatment, and provision of patient advice for the conditions. Further evaluation of the epidemiological patterns of dry eye disease prevalence has potential to inform the development of more cost-effective population-based preventative strategies to reduce the global burden of dry eye disease.

## Figures and Tables

**Table 1:** Systemic associations of dry eye disease [2,20,68,69,70,71,72,73,74,75].

Systemic Conditions
Androgen deficiency Anxiety Atopy Benign prostate hyperplasia Chronic pain condition, including back pain and other pain syndromes Cicatricial pemphigoid Connective tissue disease Depression Diabetes Ectodermal dysplasia syndrome Fibromyalgia Hematopoietic stem cell transplantation Irritable bowel syndrome Menopause Migraine headaches Parkinson’s disease Pemphigoid Polycystic ovarian syndrome Psoriasis Rosacea Sjögren syndrome Sleep disorders Stevens-Johnson syndrome Thyroid disease Toxic epidermal necrolysis Turner syndrome
**Systemic medications**
Anti-androgens Antidepressants Antihistamines Anxiolytics Hormone replacement therapy Isotretinoin
